# Understanding Patterns of Social Support and Their Relationship to an ART Adherence Intervention Among Adults in Rural Southwestern Uganda

**DOI:** 10.1007/s10461-016-1559-7

**Published:** 2016-09-26

**Authors:** Esther C. Atukunda, Angella Musiimenta, Nicholas Musinguzi, Monique A. Wyatt, Justus Ashaba, Norma C. Ware, Jessica E. Haberer

**Affiliations:** 1 Global Health Collaborative, Mbarara University of Science and Technology, Mbarara, Uganda; 2 Global Health and Social Medicine, Harvard Medical School, Boston, MA USA; 3 Massachusetts General Hospital, Center for Global Health, Boston, MA USA

**Keywords:** Social support, Adherence, Relation dynamics, ART, Uganda

## Abstract

SMS is a widely used technology globally and may also improve ART adherence, yet SMS notifications to social supporters following real-time detection of missed doses showed no clear benefit in a recent pilot trial. We examine the demographic and social-cultural dynamics that may explain this finding. In the trial, 63 HIV-positive individuals initiating ART received a real-time adherence monitor and were randomized to two types of SMS reminder interventions versus a control (no SMS). SMS notifications were also sent to 45 patient-identified social supporters for sustained adherence lapses. Like participants, social supporters were interviewed at enrollment, following their matched participant’s adherence lapse and at exit. Social supporters with regular income (RR = 0.27, P = 0.001) were significantly associated with fewer adherence lapses. Instrumental support was associated with fewer adherence lapses only among social supporters who were food secure (RR = 0.58, P = 0.003). Qualitative interview data revealed diverse and complex economic and relationship dynamics, affecting social support. Resource availability in emotionally positive relationships seemingly facilitated helpful support, while limited resources prevented active provision of support for many. Effective social support appeared subject to social supporters’ food security, economic stability and a well-functioning social network dependent on trust and supportive disclosure.

## Background

High levels of antiretroviral therapy (ART) adherence are critical for HIV viral suppression and general health outcomes. Social support has been linked to improved medication adherence and quality of life among HIV-positive patients taking ART in many settings, noteworthy in sub-Saharan Africa, whose population accounts for >90 % of prescribed ART [1–4]. Multiple mechanisms may be involved in effective social support for medication adherence. Emotional support, for example, facilitates a positive state of mind and directly improves self-efficacy to adhere [5–7]. Instrumental support, especially after ART initiation, also helps patients to sustainably overcome many other barriers to adherence. This support may involve income generating activities to overcome food insecurity, transportation challenges and other structural barriers to ART [1].

Social support, however, is not always positive. Socio-cultural, socio-economic, and relationships dynamics affect the type, pattern and level of social support provided for medication adherence in different settings [6, 8]. Negative relationships, for example, may be powered partly by the absence of instrumental support, or by non-supportive or loose family ties that affect patients’ general well-being and adaptive coping mechanism against HIV-related stigma [7, 9]. Negative effects can also be influenced by a combination of poor individual attitude or lack of social motivation to evaluate or comply with significant other’ wishes to adhere [10].The effectiveness of social support may be significantly diminished by the lack of holistic intra-, inter- and structural multi-level support aimed at complete alleviation of the disease-specific stigma that undermines ART adherence soon after initiation of treatment [11].

SMS is a widely used technology globally, including sub-Saharan Africa, and has the potential for engaging social support systems to improve ART adherence [12]. Although findings have been mixed, no SMS have been used for social support networks. In a recent pilot randomized controlled trial involving real-time adherence monitoring, multiple forms of SMS were assessed for their impact on ART adherence. Individuals receiving scheduled daily and then weekly SMS reminders at ART initiation, followed by SMS only for missed doses, were found to improve adherence compared to participants receiving no SMS reminders [13]. The addition of SMS notifications to pre-identified social supporters, which has not been explored before, provided no clear benefit to adherence. In this paper, we combined quantitative and qualitative methods to examine the individual characteristics and social-cultural dynamics that may explain the kind of social support and adherence trends in the above-noted study.

## Data and Methods

### Study Design

The pilot randomized controlled trial to assess SMS interventions to support ART adherence was conducted in a publically funded and operated hospital in Mbarara, a rural resource-limited district located in southwestern Uganda. A complete description of the study has been published elsewhere [13]. In brief, at ART initiation, 63 adults (called “study participants”) received a real-time adherence monitor (Wisepill Technologies, Cape Town, South Africa) that records and transmits over cellular networks a date-and-time stamp with each opening as a proxy for medication ingestion. They were then randomized into one of three study arms:

### Scheduled SMS

Study participants received an SMS reminder daily for 1 month and then weekly for the next 2 months. During the next 6 months, they received an SMS reminder if a cellular signal from the real-time adherence monitor was not received within 2 h of the expected dosing time, and an SMS notification was sent to one to two social supporters if no signal was received for >48 h.

### Triggered SMS

For all 9 months, study participants received an SMS reminder if a signal was not received within 2 h of the expected dosing time. For the last six, an SMS notification was sent to one to two social supporters if no signal was received for >48 h.

### Control

Participants neither received any SMS reminders nor identified any social supporters.

Noteworthy, social support intervention was similar for both intervention groups. Study visits occurred at 3 and 9 months and included socio-behavioral questionnaires and HIV viral load assessment.

### Recruitment and Enrollment of Social Supporters

Study participants were asked to identify one or two individuals from their existing social support network with whom they have had stable, long-term relationships and who were likely to be available to help them during the 9 month study follow-up period. Social supporters were eligible to participate if they were ≥18 years of age, lived within 20 km of Mbarara, owned a cell phone for personal use with reliable cellular phone reception, knew the study participant’s HIV status and reported having provided social support to the study participant at least once previously. Social support was defined as: (1) enabling the study participant to get to clinic through monetary support, direct transportation, or taking care of daily activities while he/she is absent and/or (2) motivating the study participant to take medicines (ART or otherwise), including addressing cognitive and behavioral barriers, such as depression and alcohol use. Potential social supporters were excluded from the study if they were unable to use SMS, unwilling to receive the SMS notifications, or had a severe mental condition.

Potential social supporters were contacted during the 2 weeks prior to the social support intervention period to ensure an ongoing relationship with the study participant before their enrollment. The social supporter participant was then informed that he/she may receive real-time SMS notifications during Months 4 and 9 of the study (also referred to as the social supporter intervention period) when no cellular signals were received from the study participant’s real-time adherence monitor >48 h, thus potentially indicating a lapse in adherence. Social supporters were specifically told that the number of notifications would depend on the study participant’s adherence, as well as technical function of the device, SMS platform, and cellular networks. No instructions or recommendations guiding social supporters on how to respond to the SMS notifications were given because the intervention was designed to build on existing supportive relationships of study participants.

### Study Procedures and Data Collection

All data collection was performed in the local language, Runyankole. Quantitative questionnaire data were collected from social supporters at enrollment on the following topics: socio-demographics, depression, health [14], food insecurity [15], alcohol use [16], HIV stigma [17] and social support [18]. Reports of social support received by study participants did not specify the source (i.e., social support could have occurred from outside the dyad studied here). A similar questionnaire was administered to all social supporters at exit, with addition of other closed and open-ended questions that explored their specific role in study participant’s life, relationship dynamics with study participant during study period, communication/contact with study participant and what things they do together, the type of voluntary and requested help or support presently given to the study participant towards adherence, challenges and experiences to social support, and understanding of and responses to the intervention SMS notifications.

In addition to questionnaires administered at exit, ten social supporters were purposively selected for an in-depth qualitative interview based on the study participant’s explanation for the lapse, social support characteristics, and variations in the types of social support provided. Qualitative interviews with social supporters were carried out within 2 weeks of their respective study participant’s lapse. These interviews explored selection of social supporters, type of social support, likes or dislikes of the SMS notifications, awareness of study participants’ missed dose and their response to the SMS notification. Qualitative interviews with study participants have been reported elsewhere [19].

### Data Analysis

To assess participant and social supporter correlates of poor adherence, we fit cross sectional univariable and multivariable Poisson regression models. The dependent variable was computed for each participant as the number of 48-h interruptions in medication adherence compiled between study Months 4 and 9. The multivariable model comprised of those variables whose P value was <0.2 from the univariable model. Study arm was included in all regression models to control for any potential differences in adherence owing to the adherence intervention. Only study participant gender was unequally distributed across the trial study arms, but was not associated with adherence and was not controlled for in the regression models. The study participant’s social support and the social supporter’s characteristics (i.e., type of relationship, income status, alcohol use, gender, involvement in community support group and HIV status) were selected as predictors of adherence based on their potential impact on the relationship and social support for the study participant. Study participant social support was divided into instrumental (physical and economic) and emotional (emotional and informational) support. Interactions between instrumental support and food insecurity were assessed, because the low resource nature of this setting may impede the ability to provide support despite the intention to do so. The household food insecurity access scale (HFIAS) was calculated as recommended [20], and the median score was considered as a cut-off for food insecurity. Adherence data was only censored for monitor openings by staff. Data analysis was conducted in STATA version 13 (Statacorp, College Station, Texas, USA).

Qualitative analysis was inductive and categories were derived from the social supporter interviews and open-ended questions from all social supporter questionnaires. These responses were transcribed into English and coded using NVivo10 software (Melbourne, Australia). Coded data were sorted to identify themes (i.e., repeated patterns in the data). Categories were then developed to describe the identified themes emerging from the coded data. These categories are presented with illustrative quotes from social supporter data to explain the quantitative data trends on social support by describing perspectives on type and reasons for social support, perspectives and experiences to SMS notifications, type of relationships with study participants and barriers encountered over the study period that could have affected the quality of social support.

## Results and Findings

### Participant Characteristics

A total of 21, 20 and 22 study participants were randomized into the schedules SMS, triggered SMS, and control arms, respectively. Their characteristics are presented in detail elsewhere [13] and summarized in Table 1. In brief, 65 % of study participants were female. Median age was 30 years (inter-quartile range [IQR] 25–35). Sixty-six percent had a primary school education or less. Both depression and food insecurity were common amongst study participants (48 and 37 %, respectively). Stigma was moderate with a median score of 3 (IQR 2–5) on an eight-point scale, and hazardous drinking was seen in 23 %. Reported social support was moderately high at a median score of 3.1 (IQR 2.8–3.4) on a four-point scale. The median CD4 count was 309 cells/mm^3^ (IQR 231–397).

**Table 1:** Demographic characteristics of participants and their social supporters

Characteristic	Study participants (n = 62)	Social supporters (n = 41)
Female gender, n (%)	40 (65)**	28 (68)
Median age (IQR)	30 (25–35)	34 (31–46)
Education level, n (%)		
>Primary	21 (34)	20 (49)
≤Primary	36 (58)	18 (44)
None	5 (8)	3 (7)
Able to read english or Runyankole	60 (97)	45 (100)
Median CD4 cell count (cells/mm^3^)	309 (231–397)	N/A
Regular income (yes)	11 (18)	6 (15)
Use of alcohol	26 (42)	12 (29)
Mean length of relationship in years (SD)	12.9 (12)	13.1 (13)
Tested for HIV	62 (100)	39 (95)
HIV status		
Positive	62 (100)	20 (49)
Negative	–	16 (39)
Unknown	–	5 (12)
On ART	62 (100)	20 (49)
Previous care experience for another HIV-positive person	–	25 (61)
Knowledge about HIV	62 (100)	41(100)
Community membership in community group	6 (10)	24(59)
Severe food insecurity	23 (37)	12 (29)
Depression	30 (48)	1 (2.4)
Hazardous alcohol use	14 (23)	6 (15)
Median social support score^a^ (IQR)	3.1 (2.8–3.4)	3.1 (2.6–3.6)
Stigma Score^b^	3 (2–5)	0 (0–1.7)
Median number of people providing any kind of support (IQR)	10 (5–16)	10 (5–10)

** All demographic characteristics were comparable except for gender, which was significantly different between study participant arms. Scheduled SMS arm: 15 (71 %), triggered SMS arm: 7 (35 %), control arm: 18 (86 %), P = 0.03)

^a^This score ranges from 1 to 4, with 4 indicating high levels of social support

^b^This score ranges from 1 to 8, with 8 indicating high levels of stigma

All participants in Scheduled SMS and Triggered SMS groups identified at least one social supporter. Forty-eight individuals were identified as social supporters, of whom 45 enrolled; the three ineligible individuals lived >20 km from Mbarara. One social supporter, whose study participant later tested negative for HIV, was dis-enrolled. For the three study participants with two social supporters each, the most preferred social supporter (per the study participant’s report) was considered for this analysis, leaving a total of 41 social supporters. Sixty-eight percent were female, and the median age was 34 years (IQR 31–46) (Table 1). Fifty-six percent had education levels of primary school or less, and 13 % reported problem drinking. Of the 88 % of social supporters who had tested for HIV, 56 % were positive and all attended an ART clinic. More than half (63 %) belonged to a community group and two-thirds had previously cared for an HIV-positive person. Thirty percent of social supporters reported severe food insecurity; depression was rare and stigma was scored low. Social supporters’ own social support was moderately high with a median score of 3.1 (IQR 2.6–3.6) on a four-point scale.

### Relationship Type and Quality of Social Support

As shown in Table 2, 42 % of social supporters were a spouse, 34 % were “other family” (i.e., siblings, child, parent, in-laws), and 22 % were friends. Fifty-three percent of social supporters communicated with study participants more than once a week, while 12 % never communicated with them in the last 6 months of the study (i.e., the social supporter intervention period). Although 88 % of the social supporters generally liked receiving SMS notifications, 40 % of these were responded to. Sixty-eight percent of social supporters perceived the support they provided to their respective study participants as helpful, while 32 % perceived their support as discouraging. Fifty-four percent indicated they were happy in their relationships with their study participants at exit, while 24 % were not. Slightly over a third of social supporters (39 %) reported that their relationship had improved over the course of the study, while a quarter (24 %) said it had declined; the remainder was neutral.

**Table 2:** Patterns, awareness and relationships of social supporters with study participants

Characteristic	Frequency (%)
Social supporter characteristics	
Social support relationship to the study participant	
Friend	9 (22)
Neighbor	1 (2)
Other family	14 (34)
Spouse	17 (42)
Previous care experience for another HIV-positive person	
Yes	25 (61)
No	16 (39)
Perceived HIV-positive status influence towards type of support	17 (85)*
Experience during the study	
Typical communication with the study participant during study	
More than once a week	20 (53)
Once a week	9 (24)
Less than once a week	7 (18)
Never	2 (5)
Response rate to SMS notifications (Total notifications sent = 563)	
Yes	229 (41)
No	29 (5)
I don’t remember	305 (54)
Social supporter perception of support provided to study participant	
Perceived support as helpful	28 (68)
Perceived support as discouraging	13 (32)
Social supporter likeness of SMS notifications	
Very much liked	24 (59)
Liked	12 (29)
Not liked	0 (0)
N/A	5 (12)
SS awareness on participants missed dose	10 (24)
Impact of study experience on the relationship	
Relationship satisfaction	
Extremely unhappy	4 (10)
Fairly unhappy	3 (7)
A little unhappy	3 (7)
Happy	6 (15)
Very happy	17 (41)
Extremely happy	8 (20)
Interest in relationship**	
I want desperately for the partnership to succeed &would go almost any length to see that it does	20 (49)
It would be nice if my partnership succeeded, but I can’t do much more than I am doing now to help it succeed	4 (10)
I want very much of my partnership to succeed and will do all I can to see that it does	6 (15)
It would be nice if my partnership succeeded, but I refuse to do any more than I am doing now to keep it going	3 (7)
I want very much for my partnership to succeed and will do my fair share to see that it does	3 (7)
My partnership can never succeed, and there is no more that I can do to keep it going	5 (12)
Relationship quality after participant after the study	
Improved	16 (39)
No change	15 (37)
Declined	10 (24)

* For those who reported being HIV-positive

** Adapted from the Relationship Satisfaction Scale [24]

The types and extent of social support as reported by both the study participants and the social supporters varied (Table 3). Nearly half of study participants obtained economic support with money (44 %) and transport (40 %) as much as they liked. Social supporters similarly reported providing support with money (50 %) and transport (45 %) as much as they liked. However, more discrepancies in the reported support were seen in perspectives for physical support. Half of study participants got help with housework, while 36 (62 %) received help when sick as much as they liked. On the other hand, 21 and 34 % social supporters provided help with housework and when sick as much as they liked respectively. There were also more discrepancies in reported emotional support. More than half of study participants were visited or cared for, talked about work and personal problems and got advice as much as they liked. A higher number of study participants (83 %) reported obtaining love and affection as much as liked than that offered by social supporters (76 %). Less than 50 % of social supporters talked about personal or work problems as much as they liked, while greater than 60 % provided love and affection, visited or cared for study participants as much as they liked.

**Table 3:** Social support as provided by the Social Supporters and received by participants

Type of social support, n (%)	Received by study participants (n = 62)*	Provided by social supporters (n = 41)
Instrumental support		
*Economic*		
Help with money		
As much as liked	29 (50)	17 (44)
Less than liked	23 (40)	9 (24)
Much less than liked	2 (3)	3 (8)
Never	4 (7)	9 (24)
Help with transport		
As much as liked	26 (45)	15 (40)
Less than liked	27 (47)	7 (18)
Much less than liked	1 (2)	2 (5)
Never	4 (6)	14 (37)
*Physical*		
Help with participants’ housework		
As much as liked	29 (50)	8 (21)
Less than liked	21 (36)	6 (16)
Much less than liked	5 (9)	3 (8)
Never	3 (5)	21 (55)
Help when participant is sick		
As much as liked	36 (62)	13 (34)
Less than liked	20 (35)	11 (29)
Much less than liked	0 (0)	2 (5)
Never	2 (3)	12 (32)
Emotional support		
*Emotional*		
Visit or care		
As much as liked	32 (55)	25 (66)
Less than liked	19 (33)	5 (13)
Much less than liked	4 (7)	2 (5)
Never	3 (5)	6 (16)
Love and affection		
As much as liked	48 (83)	29 (76)
Less than liked	8 (14)	3 (8)
Much less than liked	2 (3)	2 (5)
Never	0 (0)	4 (11)
Personal problems		
Talk as much as liked	33 (57)	16 (42)
Less than liked	20 (34)	13 (34)
Much less than liked	4 (7)	2 (5)
Never	1(2)	7 (19)
Work problems		
Talk as much as liked	32 (55)	17 (45)
Less than liked	21 (36)	10 (26)
Much less than liked	3 (5)	3 (8)
Never	2 (4)	8 (21)
*Informational*		
Give useful advice		
As much as liked	34 (59)	23 (61)
Less than i would like	20 (34)	4 (11)
Much less than i would like	3 (5)	5 (13)
Never give useful advice	1(2)	6 (15)

* Analysed for exit interviews

### Effects of Social Supporter Characteristics, Relationship Dynamics, and Social Support on Adherence Lapses

The average number of >48-h adherence lapses during the trial was 6.1 (SD = 5.9) per participant. When considering the univariable models, adherence lapses were less frequent when social supporters were female (RR = 0.66, P < 0.001) and had a regular income (RR = 0.28, P < 0.001) (Table 4). Adherence lapses were more frequent when social supporters were depressed (RR = 1.53, P = 0.001), had daily contact with study participants (RR = 1.55, P = 0.002) and reported food insecurity (RR = 1.79, P < 0.01). No effect was seen with the overall social support score as reported by study participants (RR = 1.21, P = 0.18) nor with instrumental (RR = 1.08, P = 0.425) or emotional support (RR = 1.19, P = 0.180) sub-scores. In the multivariable model, a regular income for the social supporter was associated with fewer adherence lapses (RR = 0.27, P = 0.001), whereas daily communication (RR = 1.8, P = 0.004) and overall social support (RR = 1.65, P = 0.009) were associated with more adherence lapses.

**Table 4:** Possible facilitators/barriers to adherence (Poisson regression)

Characteristic effect estimate	Univariable analysis		Multivariable analysis	
	RR	P	RR	P
Social supporter characteristics				
Age				
21–30	Ref			
31–33	1.01 (0.72. 1.41)	0.940		
34–45	0.72 (0.50, 1.03)	0.075		
>45	0.79 (0.56, 1.13)	0.210		
Female (yes/no)	0.66 (0.51, 0.85)	0.001	1.23 (0.81, 1.84)	0.330
>7 primary education (yes/no)	1.09 (0.85, 1.41)	0.520		
Harzadous alcohol use (yes/no)	1.14 (0.81, 1.61)	0.450		
Spouse (yes/no)	1.08 (0.83, 1.40)	0.570		
Family (yes/no)	1.08 (0.83, 1.40)	0.581		
Friend (yes/no)	0.75 (0.53, 1.06)	0.111	1.68 (0.98, 2.87)	0.059
Daily communication (yes/no)	1.55 (1.17, 2.05)	0.002	1.78 (1.20, 2.64)	0.004
Regular income (yes/no)	0.28 (0.15, 0.51)	<0.001	0.27 (0.12, 0.59)	0.001
HIV-positive (yes/no)	1.30 (0.97, 1.73)	0.081	1.04 (0.72, 1.49)	0.830
Depression	1.56 (1.20, 2.03)	0.001	1.04 (0.68, 1.58)	0.860
Stigma (yes/no)	1.1 (0.86, 1.41)	0.450		
Food insecurity				
No (HFIAS ≤8)	Ref		Ref	
Yes (HFIAS >8)	1.79 (1.35, 2.36)	<0.001	1.05 (0.01, 0.59)	0.018
Community support group (yes/no)	0.85 (0.64, 1.13)	0.26		
Participant characteristics				
Reported social support (overall)	1.21 (1.02, 1.74)	0.180		
Instrumental support	1.08 (	0.425		
Emotional support	1.19	0.180		
Interactions				
Instrumental support				
Food secure (HFIAS ≤8)	0.58 (0.40, 0.82)	0.003	0.48 (0.27, 0.87)	0.015
Food insecure (HFIAS >8)	1.34 (1.06, 1.71)	0.017	1.53 (1.10, 2.13)	0.011

All models are adjusted for study arm

Because social supporters’ food insecurity was highly significant, explained the most variation (7 %) in the models compared to other variables, and could interfere with the ability to provide instrumental support, we explored an interaction between these two variables. We found that instrumental social support was associated with an increased number of adherence interruptions (RR = 1.34, P = 0.017) among social supporters reporting food insecurity, while instrumental social support among those not reporting food insecurity was associated with a reduced number of adherence interruptions (RR = 0.58, P = 0.003; interaction term P < 0.001).

### Qualitative Results

Social support for study participants was diverse and dynamic. A total of seven categories were identified from the interviews and are presented here: (1) Concern for disclosure and social support; (2) Dysfunctional relationships and the impact on social support; (3) Physical barriers and financial difficulties; (4) Food insecurity preventing social support; (5) Daily contact affecting positive social support; (6) Alcohol use and response to notifications; and (7) Study role awareness and response to SMS notifications (study intervention).

### Concerns for Disclosure and Social Support

Social supporters’ perspectives of why they were chosen by study participants included two major considerations: awareness of the study participants’ HIV-positive status and trust in their ability to keep it a secret from people in the study participants’ life and the community. These factors seemed to be more important than the actual provision of prior support. The fear of disclosure and the expectation for social supporters to keep their HIV-positive sero-status confidential seemed to limit the number of people who could help offer support to study participants.

I am the only person who knows she is HIV-positive and she begged me not to tell anyone, not even our other family members. She fears other people will look down on her, blame her, judge her or mock her if they know her status…She has no other alternative so it’s very important I keep it secret. (study participant’s brother).

Additionally for some study participants, social supporters were close relatives and friends who were not only trusted and able to provide help, but also seemed to understand their plight. According to a study participant’s sister,

At first, she feared for our family and other people to know her HIV status. She thought they would not accept her and like blame her…However, I am her big sister and she trusts me. I virtually help her with everything: food, shelter, money, school fees for her children, ever since her husband abandoned her. We are very close.

She trusts me with her secrets and I promised to keep them…At least she is at peace telling me everything and I understand why she would not want her husband or sisters or brothers to know about her status yet. I understand her and being HIV-positive myself, I can’t judge her unfairly and I wouldn’t disclose her status just like that. (study participant’s female friend).

### Dysfunctional Relationships and the Impact on Social Support

Generally, qualitative data showed positive relationships between study participants and social supporters of different gender and particularly where the two were not married partners. **However, s**ome social supporters stated that their relationships stalled or became turbulent over the study period. This relationship turbulence was characterized by trust disintegration, unsupportive behavior, stigma and fear of disclosure, victim blaming, suspicions of infidelity and emotional blackmail through status disclosure to others that often times ended in communication breakdown, misunderstandings, resentment, separation or relationship dissolution. The turbulence was often due to ongoing relationship complications arising from feelings of distrust and betrayal and not related to the intervention itself.

I have asked her where she got the (HIV) virus and she lied to me that she was born with it… I know I am negative and that (HIV) is her problem… I don’t care (whether she takes her pills or not). I can’t even sleep with her because she is sick (HIV). She is a liar. I am not a fool…We don’t talk these days and I don’t trust her anymore. I plan to tell her parents (about her HIV status) and I know she is very scared. (Study participant’s husband).

A study participant’s wife added, He is full of himself. I don’t trust him anymore…He is ungrateful and is not even bothered on how I feel about how we got here (being HIV-positive) in the first place because of him…We have had many fights because he suspects me of telling some of his friends about his status so I left him…It’s been a while. I don’t call nor talk to him anymore and truthfully, I am not bothered.

Some social supporters thought study participants felt obligated not to disappoint them which thereby facilitated adherence, especially when they ably and continuously provided or cared for the study participants.

I am her elder sister and I virtually provide for her and her children everything. She fears me a lot because she knows I will not be happy with her if she hides anything from me like if she is not taking her medications seriously as prescribed (study participant’s sister).

Additionally, social supporters perceived a sense of ‘shared knowledge’ on pill-taking behaviour through SMS triggered after missed doses. Even so, their relationship seemed to get strained over time when social supporters perceived study participants as continuing to miss their doses or social supporters’ assumptions about reasons for missing doses. Misunderstandings for example, often arose as a result and social supporters felt resentment towards study participants. These feelings of resentment seemed to further affect the social supporters’ general response to the SMS notifications (the intervention), especially from those resenting that HIV had come into their lives.

According to one study participant’s wife, Of course I was able to know when he is missing his pills when I got the SMS and I was happy… however, when I saw the reminders recently, I did not care to ask him about it nor his pills. I got tired of helping someone who doesn’t care about my life too. He sometimes doesn’t come back home and I think he hasn’t learnt from his past mistakes of sleeping around like that.

Another study participant’s husband added, At least I get to know when she is not taking her pills whenever I receive these SMS [silence]… but we have been fighting lately and she makes excuses to run away to clubs and sleep out with friends and makes up stories why she didn’t return home to take her pills…she doesn’t care. She is ungrateful and doesn’t listen to me anymore. She always shouts at me whenever I pester her to take her pills and says I don’t understand her so I have decided to just see them (notifications) and keep quiet until she grows up.

### Physical Distance and Financial Difficulties

Physical distance between the social supporter and study participant seemed to affect social support. Some social supporters reportedly experienced major challenges that impeded their ability to provide close and continued support to study participants as desired. This situation arose when study participants travelled or were out of reach due to cellular network problems, dead phone batteries, or stolen phones. In some instances, social supporters felt that study participants were simply unable to return home regularly or get in touch with them for a very long time due to cellular network problems or financial difficulties experienced after travelling. Additionally, some social supporters attributed some of the skipped pill doses, missed ART refills or clinic review visits to their inability to directly or financially help these study participants. This support was perceived to have facilitated the study participant to get their meals, return home or go to the clinic in time.

She sometimes travels to the village to do some work for some weeks and transportation there is very poor…One time, she forgot her device and pills with me. I tried calling her several times but her phone was not going through for days and yet she was unable to return in time because she had no enough money of her own for transport…I also couldn’t help her because I had no money (study participant’s female friend).

Sometimes, he travels to look for work to support us for many weeks and sometimes I don’t know what to do for days because his phone always has battery and network problems… His boss also takes so long to pay him his wages and sometimes goes for weeks without any money for food [silence]. I can’t help him with any food we grow here because he’s far away. I also don’t have a job so obviously, I can’t help him with money for transport back home in time for his clinic refills (study participant’s wife).

### Food Insecurity Preventing Social Support

Another major challenge to providing consistent and desired social support seemed to stem from social supporters’ food insecurity because of irregular resources. This situation was perceived to lead to missed doses especially when the study participants reportedly told social supporters that they could not take the ‘hard pills’ on an ‘empty stomach’. The sense of obligation felt by social supporters to help the study participant and their fear that their inability to provide food or other desired support regularly contributed to poor adherence. It sometimes resulted in misunderstandings or feelings of a burdened relationship especially if the social supporter was the sole provider of the study participant. According to one of the study participant’s brother,

She often tells me she can’t take the hard pills [ART] on an empty stomach without porridge or a good meal at super but what can I do? I know she sometimes uses it as an excuse and this upsets me…I have been having some financial challenges ever since I lost my job, which she does not seem to understand.

Town life is very difficult. We buy everything; food, fees, rent and others…I know its my responsibility to provide for her but we only could afford one small meal a day. She was unhappy with me when it became difficult for me to provide for her special meals, shelter, transport and other needs when I have no job at all so she left to stay with another relative. (study participant’s paternal aunt).

### Daily Contact Affecting Positive Social Support

Social supporters reported that their efforts to support study participants sometimes seemed to be experienced negatively. The regular contact and closeness that facilitated their ability to provide support sometimes seemed to be unhelpful to study participants, especially when social supporters thought their support was perceived to be unsupportive or confrontational by study participants in instances of a missed dose. The intolerance and communication breakdown was often times exacerbated by ongoing misunderstandings that seemed to trigger feelings of nagging, overbearing, resentment, frustration and victim blaming mainly from family members staying close or living with the study participants, with social supporters opting to stay away.

He shouts at me for constantly asking him about his medicines everyday so I stopped asking about them. He doesn’t listen to me at all and says I nag him… (study participant’s wife).

I have not asked him about the recent ones (SMS notifications) because many times he told me I was annoying him, bugging him and following him around to tell him what to do as if he is a small boy, but the counselor told me we would both die if he doesn’t take his medicines seriously on time… (study participant’s wife).

### Alcohol Use and Response to SMS Notifications

Alcohol use amongst some social supporters seemed to explain the poor quality of support that was given to some study participants at the time of an adherence lapse. Whereas some social supporters with regular alcohol use reported not seeing the SMS notifications at the time they were sent, others forgot, delayed or were uninterested in following up to intervene or help support the study participant especially in complicated relationships (e.g., sero-discordancy or communication breakdown) between the social supporters and the study participants.

I am often drinking [alcohol] with my friends in the evenings and I return home late. Sometimes, I am very drunk and get to see these messages the following day when it’s of no use. Besides, taking pills is her own business and not mine. (study participant’s husband).

Another study participant’s wife added, Sometimes I go out and drink [alcohol] with our neighbors and it gets difficult to keep track of time [for medication] or see your messages [notifications] until the next day or so when I can no longer talk to him or he has travelled.

### Understanding the Study Role and Purpose of the SMS Notifications (Study Intervention)

The social supporter’s knowledge of the purpose of SMS notifications (the study intervention) and the importance of adherence played key roles in promptly responding or contacting study participants at the time of the SMS notification.

I care about him a lot. You see, I get to know when he is taking his medicines and when he is not through these messages I receive. I therefore try and help him in any way I can to make sure he takes his medicines on time so he gets better and take care of his young children, (study participant’s brother in-law).

On the contrary, some participants did not seem to understand the intended purpose of the SMS interventions, thus limiting the potential for impact.

I think the messages were meant to help me know about his clinic dates and probably remind him to go to the clinic to pick more pills…so I would wait whenever we met, I would just ask him if he still has his pills and that’s all. I wouldn’t ask how and when he takes his pills because that’s not my job. (study participant’s male friend).

In summary, although a well-functioning social network built on trust, understanding, tolerance and supportive behavior seemed to have played a key role in providing useful social support to some of these newly diagnosed HIV-positive individuals, useful social support was not given to many of them. Social supporter’s inability to provide consistent and desired social support stemmed not only from challenges of physical distance and limited communication from each other, but also from food insecurity and irregular resources to actively help study participants access their meals, ARVs or travel to clinic for reviews or pill refills in time. Additionally, dysfunctional relationships involved trust disintegration, unsupportive behavior, stigma and fear of disclosure, victim blaming and suspicions of infidelity, all seemed to have a major effect on social support and medication-specific adherence.

## Discussion

In a pilot randomized controlled trial to assess the impact of SMS interventions on ART adherence in southwestern Uganda, SMS notifications of adherence lapses sent to social supporters showed no clear benefit for the adherence of the HIV-positive individuals taking ART. This analysis of social supporter characteristics and the relationships between social supporters and the HIV-positive individuals taking ART found that social supporters with a regular income and food security were significantly associated with decreased frequency of adherence lapses, whereas daily communication between social supporters and the individuals taking ART was associated with increased frequency of adherence lapses. Qualitative interview data revealed diverse and dynamic relationships. Social supporters with resources in emotionally positive relationships seemed to provide good support; however, complex relationship dynamics prevented the provision of support for many. Additional barriers to the SMS notification intervention included heavy alcohol use by the social supporter, physical distance between the social supporter and the HIV-positive individual and technical difficulties with cell phones.

This analysis highlights the complexity of social support and ART adherence. While the median level of reported overall social support was high, instrumental support was only beneficial when the social supporter had food security. The importance of available resources for improving adherence in a resource-limited setting is not surprising. Socio-economic factors and economic stability have been documented as key facilitators to ART adherence [8]. The social supporters’ lack of resources seemed to limit their ability to actively provide instrumental help to study participants to access basic needs like food, medicines or transport to the clinic in time. It also seemed to affect the value or functionality of physical contact whenever such lack of resources forced study participants to travel to far places for many days in search of jobs to support themselves and their families. This lack of close and consistent supportive ties with social networks coupled with other interrelated socio-economic factors, like depression, stigma and food insecurity, affects the active provision of social support. The challenge is especially high when people are unable to sustainably overcome the barriers embedded in not only food insecurity, but also inability to generate regular incomes to sustain their basic economic needs [15, 21]. Although the study attempted to address some of these issues by including only social supporters who provided support in the past, food security appears to play a key role in the quality of support provided and warrants further assessment in future iterations of this type of adherence intervention.

The negative effect observed with social supporters’ daily contact could have been as a result of feelings of burden, nagging or resentment particularly if study participants felt they were being victimized or judged unfairly within their social networks. This finding suggests that contact alone does not indicate a relationship capable of promoting ART adherence. The qualitative data also indicate the importance of the specific dynamics within the relationship between the social supporter and HIV-positive individual. Misunderstandings, frustration, communication breakdown, and resentment were common and led to problems such as emotional blackmail, feelings of ungratefulness, mistrust and separation. Previous work has also shown that not all social support is helpful for medicine-specific adherence for HIV, as well as other chronic conditions in diverse settings. A good sense of support seems to be affected by the lack of close, consistent and supportive relationships with friends and family [3, 22]. These malfunctioning relationships and complexities within social networks have further been associated with poor quality support that diminishes positive effect on medicine-specific adherence [6, 23].

Relationship problems reflected the stresses of HIV infection and recent HIV diagnosis, including stigma, as well as routine relationship dynamics that can happen within any dyad. The SMS notification, however, may also have served as a trigger or catalyst for relationship problems especially if social supporters’ approach was confrontational, following notifications of a missed dose. Although all study participants and social supporters joined the study voluntarily and with full informed consent, these findings suggest further screening for potential relationship challenges that would be important for future SMS notification interventions. Indeed, we found that many social supporters reported to have been chosen majorly because he or she was the only person to whom the study participant had disclosed. Efforts to expand disclosure may also be useful in identifying healthy relationships for promoting adherence support. Additionally, education and support of social supporters (e.g., making sure they understand the importance of adherence and helping them to identify effective interventions for their study participant’s specific challenges) may be useful towards building positive adherence support. Whereas our major goal in enlisting existing social supporters was to leverage community-based resources, the complexities of some relationships observed may require some degree of ongoing involvement of healthcare providers to yield desired results. However, such efforts would increase the burden of the intervention on the healthcare system, but could potentially increase effectiveness in adherence support.

Our study had a number of strengths. It was a mixed method analysis of a randomized controlled trial conducted in a publically funded and operated hospital in a rural low-resource setting. Results therefore may have applications for similar settings. There were also important limitations. Our study was a pilot, analyzing data for only 41 social supporters. The small size of this study may have limited its ability to fully explore the association between social supporter characteristics and adherence lapses. The univariable results suggested numerous other characteristics that may play a role, including older age, female gender, alcohol use, and depression. Moreover, these factors may be highly dependent on cultural settings. Larger studies in diverse populations will be needed to fully evaluate an SMS notification intervention among social supporters.

## Conclusions

Our study contributes to a greater understanding of the characteristics and complexity of social network relationships that may influence medication-specific adherence; not all reported social support is helpful. We found many study participants unable to get effective social support from the dyad being studied. Specifically, spouses did not seem to be good social supporters for SMS reminders regardless of the complexity of their relationship with study participants. Effective social support on the other hand appeared subject to well-functioning social networks and dependent on building trust, understanding and supportive disclosure, as well as debunking stigma, especially amongst recently diagnosed HIV-positive individuals. Effective instrumental social support in particular, appeared to be enabled by food security and income stability.

ART adherence interventions relying on social support therefore require contextual understanding of individual needs and factors that facilitate a helpful environment for vulnerable patients to maximize treatment outcomes. Approaches to social support training and awareness in supportive behavior should therefore be explored carefully.
